# Social Problems during Pregnancy under the Coronavirus Disease 2019 Epidemic in Japan

**DOI:** 10.31662/jmaj.2020-0109

**Published:** 2021-02-17

**Authors:** Shunji Suzuki, Masako Eto

**Affiliations:** 1 Department of Obstetrics and Gynecology, Japanese Red Cross Katsushika Maternity Hospital, Tokyo, Japan

**Keywords:** Coronavirus disease 2019 epidemic, Japan, pregnancy, social problems

Since 2016, the frequency of pregnant women with social problems, who are recognized as “specific expectant mothers, i.e., pregnant women with at least one social risk factor ^(1), (2), (3)^,” have increased significantly year by year. The breakdown of high-risk factors associated with their difficulty raising children has been almost unchanged; however, in 2020 the impact of the Coronavirus disease 2019 (COVID-19) epidemic may be too great to continue the factors. Therefore, we examined the changes in status of the social problems and our support under the COVID-19 epidemic.

The study protocol was approved by the Ethics Committee of the Japanese Red Cross Katsushika Maternity Hospital. Informed consent concerning retrospective analyses was obtained from all subjects.

In this study, we examined the frequency, associated factors, and outcomes of specific expectant mothers managed at our institute under the COVID-19 epidemic (from April to September 2020) compared with those in 2019 as reported previously ^(3)^. Data are expressed as numbers and percentages. The one-way analyses of variance were used. Differences with *p* < 0.05 were considered significant.

Table 1 shows the number (frequency) of the specific expectant mothers managed at our institute and main social factors associated with the specific expectant mothers in the two periods (duplicate). In our previous study ^(3)^, the frequency of the specific expectant mothers increased significantly from 2016 to 2019 year by year (*p* < 0.01); however, it decreased significantly under the COVID-19 epidemic (*p* < 0.01); however, the frequency of the high-risk social factors remained almost unchanged between the recent two periods.

**Table 1.** Main Social Factors Associated with the Specific Expectant Mothers in 2019 and April-September 2020 (Duplicate).

Year	2019	April-September, 2020
Total	1,650	810
Specific expectant mothers	435 (26.4)	171 (21.1)^#^
Social factor		
First visit at ≥ 15 weeks	50 (11.5)	17 (7.6)
Teenage	29 (6.7)	15 (6.7)
No regular visit	6 (1.4)	5 (2.2)
Unmarried	115 (26.4)	45 (20.0)
Unplanned pregnancy	73 (16.8)	31 (13.8)
Partner		
Teenage	12 (2.8)	8 (3.6)
Unemployed	18 (4.1)	10 (4.4)
Initiate partner violence	51 (11.7)	28 (12.4)
Economic problems		
Grant target	44 (10.1)	19 (8.4)
Nonsubsidized	26 (6.0)	17 (7.6)
Lack of support	60 (13.8)	32 (14.2)
Mental disorders	38 (8.7)	21 (9.3)
Other disorders	9 (2.1)	7 (3.1)
Positive of mental screening*	28 (6.4)	7 (3.1)
Foreigners who cannot talk	61 (14.0)	33 (14.7)
Somehow anxious	85 (19.5)	44 (19.6)

Data are presented as number (percentage). ^#^ *P* < 0.05 vs. 2019. * Positive of mental screening without risk factors of mental disorders only.

Table 2 shows the occupations supporting the social factors associated with the specific expectant mothers (duplicate) and the number of mothers unable to raise children. In our institute, the percentage of required occupations increased significantly under the COVID-19 epidemic (*p* < 0.01).

**Table 2.** Support Occupations and Mothers Unable to Raise Children in 2019 and April-September 2020 (Duplicate).

Year	2019	April-September 2020
Number of specific expectant mother	435	171
Medical social workers	125 (28.7)	82 (48.0)^#^
Clinical psychologists	78 (17.9)	24 (14.0)
Regional administrative staffs	112 (25.7)	75 (43.9)^#^
Mother unable to raise	2 (0.5)	3 (1.8)

^#^ *P* < 0.05 vs. 2019.

Based on the current results, the ratio of specific expectant mothers seemed to decrease under the COVID-19 epidemic; however, more active support was required for them. It may suggest that pregnant women in Japan may have more severe social and/or economic distress and social isolation than expected. They tend to avoid complaining about their problems on their own ^(4)^. Because this tendency has been presumed to be stronger under the COVID-19 epidemic, which restricts their behavior, their number/ratio might have decreased. Under these restrictions, pregnant women have greater social problems and we believe that an increasing number of pregnant women are in need of social support.

According to the reports from our social workers, the women who were conspicuous were as follows: foreigners without medical insurance who could not return to their countries, hospitalized women with disabilities who cannot take care of themselves under visitation restrictions, teenagers who have unexpectedly become pregnant, etc. In addition, there were some pregnant women who refused support to avoid contact with others due to the COVID-19 epidemic.

Therefore, a more proactive approach from medical institutions is needed to solve social problems of specific pregnant women under the COVID-19 epidemic.

## Article Information

### Conflicts of Interest

None

### Author Contributions

Shunji Suzuki: project development, data management, data analysis, manuscript writing/editing.

Masako Eto: project development, data collection, data analysis, manuscript writing/editing.

### Consent

The study protocol was approved by the Ethics Committee of the Japanese Red Cross Katsushika Maternity Hospital (K2019-26-2).

Patients’ informed consent for publication of this report was obtained.

## References

[1] Ministry of Health, Labor and Welfare. Parenting support visit business guideline [Internet]. [cited 2020 Dec 10]. Available from: https://www.mhlw.go.jp/bunya/kodomo/kosodate08/03.html. Japanese.

[2] Bureau of Social Welfare and Public Health. Tokyo Metropolitan Goverment: Social welfare and public health in Tokyo [Internet]. [cited 2020 Dec 10]. Available from: https://www.fukushihoken.metro.tokyo.lg.jp/english/about/pamphlet.html.

[3] Suzuki S, Eto M. Current status of social problems during pregnancy at a Perinatal Center in Japan. JMA J. 2020;3(4):307-12. 33225102 10.31662/jmaj.2020-0056 PMC7676988

[4] Suzuki S, Takeda S, Okano T, et al. Recent strategies in perinatal mental health care in Japan. Hypertens Res Preg. 2018;6(1):11-4.

